# Novel Durable Antimicrobial Ceramic with Embedded Copper Sub-Microparticles for a Steady-State Release of Copper Ions

**DOI:** 10.3390/ma10070775

**Published:** 2017-07-10

**Authors:** Adam J. Drelich, Jessie Miller, Robert Donofrio, Jaroslaw W. Drelich

**Affiliations:** 1 Department of Materials Science and Engineering, Michigan Technological University, Houghton, MI 49931, USA; ajdrelic@umich.edu; 2 NSF International, 789 Dixboro Rd., Ann Arbor, MI 48105, USA; jdmiller@nsf.org (J.M.); RDonofrio@neogen.com (R.D.)

**Keywords:** antimicrobial ceramic, copper, water disinfection

## Abstract

Using pottery clay, porous ceramic stones were molded and then decorated with copper sub-microparticles inside the pores. Copper added antimicrobial functionality to the clay-based ceramic and showed ability in disinfecting water. Populations of both *Staphylococcus aureus* and *Klebsiella pneumoniae* in contaminated water were reduced by >99.9% in 3 h when exposed to an antimicrobial stone. This antimicrobial performance is attributed to a slow release of copper into water at both room and elevated temperatures. Copper is leached by water to produce ion concentrations in water at a level of 0.05–0.20 ppm after 24 to 72 h immersion tests. This concentration is reproducible over a number of cycles >400. To our knowledge, this is the first formulation of copper sub-microparticles inside the porous structure of commercial-sized ceramic stones that can disinfect bacteria-contaminated water over a period of at least several months.

## 1. Introduction

The spread of diseases is a serious problem for the growing human population. Deaths from acute respiratory infections, diarrheal diseases, measles, AIDS, malaria and tuberculosis accounted for more than 85% of the mortality from infection worldwide [1]. Added to this is the significant global burden of resistant hospital-acquired infections, the emerging problems of antiviral resistance, and the increasing development of drug resistance in the neglected parasitic diseases of poor and marginalized populations [2]. The fight against infectious bacteria and viruses has been broadened in recent years through deployment of antibacterial personal-use products such as sprays, soaps, and tissues. However, only a small fraction of the human population uses antibacterial products due to cost, limited access, or ignorance, and therefore antibacterial sprays, soaps, and tissues cannot solve this ever-growing health problem. Additionally, new antibacterial products promote the development of resistant bacteria.

Futuristic predictions often envision a world in which architecture, facilities, commodities, and products will be engineered with smart surfaces and coatings. Such surfaces will “intelligently” respond to changing environments and/or personal needs. Those surfaces and products that are repeatedly touched in the course of daily activity, as well as those in possible contact with bacteria-contaminated water and food, currently pose a serious health threat by harboring infectious agents. These surfaces will need to be engineered to be inhospitable for bacteria, viruses, fungi, and molds. Most likely, commodities with bacteria-resistant surfaces, engineered through advances in nanotechnology, will be not only used in hospitals to prevent the worldwide spread of so-called “superbugs”, but also in schools, offices, on buses, trains, etc.

Copper and copper compounds have been shown to kill a wide range of microorganisms, including viruses (enveloped and non-enveloped), bacteria (gram positive and negative), fungi, yeast, and even spores [3,4,5,6]. In fact, according to the Copper Development Association (CDA), copper is able to kill 99.9% of harmful bacteria within two h, and to keep killing over 99% of bacteria regardless of repeated exposure to the copper surface. This is true even for antibiotic-resistant bacteria such as Methicillin-resistant *S. aureus* (MRSA) [7].

The antimicrobial property of copper is achieved through several mechanisms, which work in tandem. These include membrane lipid peroxidation, denaturation of nucleic acids, plasma membrane permeabilization, alteration of proteins, and inhibition of protein biological assembly and activity [8,9]. It is believed that copper damage begins at the microorganisms’ envelope [10,11]. Copper is also capable of interacting with microbial proteins and nucleic acids both on the envelope and within the cell [12,13,14,15]. One major mechanism for these interactions is a cyclic redox reaction between Cu^+^ and Cu^2+^, which causes the production of hydroxyl radicals capable of damaging vital components of a microorganism [8,16]. It is this redox reaction which makes copper an effective antimicrobial substance. Additionally, despite being used by human civilization for many centuries, no microorganisms have been found which are completely resistant to copper [17,18,19,20].

Copper, in general, is acknowledged to be safe for humans [21]. Common antimicrobial uses for copper include wound healing [22,23], bacteria control in hospitals [24,25,26], prevention of parasite and algae growth in water reservoirs, and reduction of foodborne diseases [27,28,29]. In recent years, new antimicrobial materials carrying small quantities of copper, typically in the form of nanoparticles or nanodots, have been developed with the purpose of replacing large quantities of copper and its alloys [30,31,32]. Synthesis of copper nanoparticles on natural aluminosilicates such as montmorillonite, kaolinite, palygorskite, clinoptilolite, and others has been broadly demonstrated experimentally as well, and these mineral-copper hybrid materials seem to be a promising alternative to bulk copper-based components [30,32].

The use of treated ceramics for water decontamination has seen some success already. Specifically, the coating or embedding of photocatalysts, such as titanium oxide (TiO_2_), into ceramic-like material has been used to treat water at an industrial scale [33,34,35]. Such treatments have been shown to be durable, remaining effective over multiple cycles, and capable of clearing pollutants and inactivating harmful bacteria such as *E. coli*. The use of ceramics and ceramic-like material is also cost-effective, as many of the materials are cheap and plentiful. The studies do suggest, however, that the composition, diameter, and other parameters of the vessel material can have profound effects on the disinfecting qualities of the product. Here, we report the formulation and antimicrobial performance of new clay-based stones. The uniqueness of this invention is in embedding copper sub-microparticles into the internal 3-dimensional structure of a porous clay-based ceramic, taking advantage of the large surface area. This new architecture allows for a release of copper ions into water in small quantities over a long period of time, making this hybrid material antimicrobial and durable. This study, although of exploratory nature, demonstrates that antimicrobial stones work effectively in both cold and warm waters and are perfectly suitable to serve as a sanitizer for small quantities of water, up to 1 gallon per stone, for non-potable applications. Such small quantities of water are typically used in SPA, beauty and horticulture industries as well as for personal hygiene in households. Specifically, the applications of this antimicrobial invention could be explored in treatment of water in SPA massaging studios, nail and pedicure salons, hair dressing parlors, and several others. It could also replace bleaching pills used commonly in toilets.

## 2. Results and Discussion

**Figure 1:**
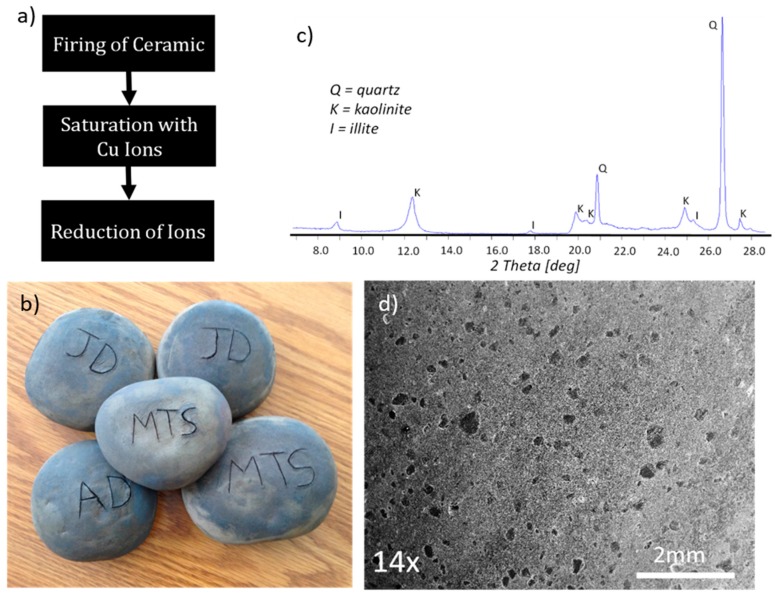
(**a**) schematic of the three steps in manufacturing antimicrobial ceramic stones; (**b**) ceramic stones; (**c**) x-ray diffraction pattern of the clay used in formulation of stones; (**d**) scanning electron micrograph of ceramic stone revealing porosity.

Antimicrobial ceramics were formulated through a three-stage process that included firing of clay-based porous ceramic, saturation of ceramic with copper ions, and conversion of copper ions into copper sub-microparticles (Figure 1a). These ceramics had dimensions of 5–6 cm and are shown in Figure 1b. Digital image analysis of scanning electron micrographs of the ceramics revealed a porosity of 16 ± 2% with channels that varied from 0.3 mm down to a fraction of a micrometer (Figure 1d). There is room to manipulate porosity through addition of organic matter that decomposes at temperatures of ceramic firing, but optimization of porosity and resulting internal surface area were not explored in this pilot applied study.

Pottery clay with kaolinite and small quantities of illite, as per XRD pattern in Figure 1c, was used in this study. Firing of the clay at 1000 °C was necessary to produce solid but porous ceramic that is stable in water. Although not studied in detail, a partial structural collapse of kaolinite and its reorganization to metakaolinite most likely occurs at firing temperature, with kaolinite dehydroxylation at 450–600 °C [36]. Additionally, starting at 950 °C, spinnel forms and transforms to mullite, producing vitrified ceramic, while the crystalline structure of illite breaks down at 700 °C [36]. X-ray diffraction analysis of the ceramic after firing confirmed the disappearance of structural kaolinite and illite (not shown). These phase transformations, however, have no major effect, in general, on introduction of antimicrobial copper ions necessary for formulation of copper sub-microparticles.

**Figure 2:**
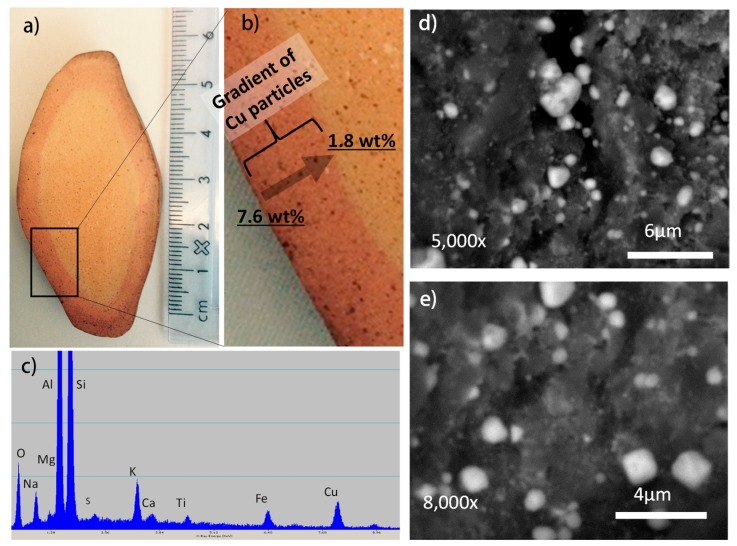
(**a**,**b**) optical microscopy images of a cross section of antimicrobial ceramic showing a zone of ceramic saturated with copper sub-microparticles; (**c**) X-ray energy dispersive spectrum for a ceramic zone saturated with copper sub-microparticles; (**d**,**e**) backscattered electron images of internal structure of ceramic decorated with copper sub-microparticles.

In the second stage of the manufacturing process (Figure 1a), ion saturation, adsorption and exchange processes were carried out on fired ceramic to introduce copper ions into ceramic structure using concentrated copper sulfate solution [37]. Clays, including kaolinite and metakaolinite, compete for ions [38,39,40,41,42,43] through the capillary penetration of copper sulfate solution into ceramic porosity, followed by adsorption on surfaces of aluminosilicates and ion exchange for clays. During the ion exchange process, copper ions replaced some of the calcium and other interlayered ions on surfaces of kaolinite/metakaolinite as confirmed with X-ray elemental analysis (not shown). The amount of copper ions introduced to the clays and other minerals is dictated by the adsorption and ion exchange capacities of aluminosilicates, solution composition (pH and ionic strength), and temperature and time of ion exchange process [30]. In porous ceramic like that used in this study, additional copper ion solution fills the fine channels of the ceramic’s porosity, making the copper ion saturation process more efficient than in cases of powdered clays. Since ion saturation of the internal structure of a ceramic is limited by diffusion of copper ions through internal porosity, this process can produce a gradient of distribution for copper ions, with higher concentration on the ceramic surface. In this study, using experimental conditions as described in the Methods, the majority of copper was introduced into the outer shell of the ceramic to a depth of 4 to 5 mm (Figure 2).

Ion-saturated ceramic can have an inherent weakness in the potential release of mobile copper ions in toxic quantities during its contact with water and moisture. The solution to this problem is the reduction of the mobile ions to immobile sub-microparticles that are embedded into the porous structure of the ceramic and strongly adhere to mineral surfaces. Conversion of cations embedded in the structure of minerals to metallic sub-micro and nanoparticles is a relatively new approach [30,44,45,46,47,48,49,50], although this concept originates from research on modification of synthetic zeolites [51,52,53,54]. Hydrogen is most commonly used to reduce cations to their elemental forms through the reaction:

(1) $$M^{2+}+H_{2}\to M^{0}+2H^{+},$$

where M is the metal. In our approach, it was done at 450 °C for 4 h. The reduced metal ions formed sub-microparticles with diameters varying from approximately 2 µm to less than 0.5 µm that covered mineral surfaces inside porosity as shown in Figure 2.

The ceramic stone was modified with copper to a depth 4–5 mm under the manufacturing conditions selected in this study (Figure 2a,b). The ceramic holds copper at a quantity of about 7 wt % in the surface region, which dropped to less than 1 wt % at a depth of ~5 mm (darker “skin” region shown in Figure 2a,b). Copper content in ceramic below the “skin” region was less than 1 wt % and often less than 0.1–0.4 wt %.

The exact mechanisms of nanoparticle formation, migration, and coarsening remain poorly understood, but our observations suggest nucleation, migration and coarsening of copper sub-microparticles on mineral surfaces does occur. Following earlier work on preparation of catalytic particles on synthetic zeolites [52,53], it is expected that the size of sub-microparticles and their distribution are affected by the reduction temperature (including calcination if included as the proceeding step), time of reduction, concentration of copper ions in the parent sample, and mineralogy/chemistry of the mineral carrier. Unfortunately, neither qualitative nor quantitative correlations are known at present, and detailed studies on mechanisms of nanoparticle formation will need to be studied in future research.

The reduction of ionic to metallic copper was accomplished using hydrogen at high temperatures with the following reaction:

(2) $${\mathrm{Cu}}^{2+}+H_{2}\to {\mathrm{Cu}}^{0}+2H^{+}.$$

The hydrogen ions formed are either attached to the aluminosilicate framework by electrostatic forces or consumed by the hydroxylation reaction at a site with local charge imbalance:

(3) $$H^{+}+A-O^{-}\to A-\mathrm{OH},$$

where O represents a lattice oxygen and A represents the aluminosilicate framework. The study with silver-saturated chabazite suggests progressive structural changes in this natural zeolite, where breaking the Si–O–Al bonds occurs [55].

Because copper atoms are no longer retained by electrostatic forces in interactions with minerals after hydrogen reduction, they migrate laterally on surfaces and form small crystallites and sub-microparticles. The driving force for this migration is probably the lattice energy of the metal combined with the copper-mineral interactions, moderated by non-specific diffusion effects. The polarity of the mineral carrier combined with geometrical restrictions of porosity could be the reason for delivery of metallic sub-micro and nanoparticles to certain locations in porous architectures [47,56]. The size of the crystallites (sub-micro and nanoparticles) formed, on the other hand, is most likely dictated by the coarsening process between atoms and crystallites and depends upon initial concentration of copper ions along with the time and temperature of reduction process. It was also observed that copper sub-microparticles were found to tightly adhere to mineral surface pores as could be judged by the shape of sub-microparticles at the base and observations that they remained attached even after several months of contact of ceramic with water (not shown).

**Figure 3:**
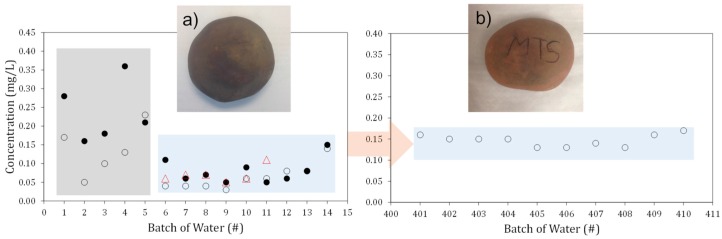

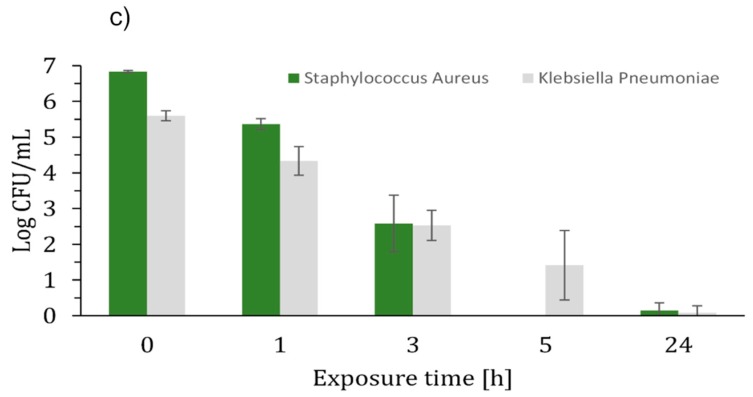
(**a**,**b**) Concentration of copper ions in water over multiple contacts of one stone with 1.9 to 3.8 L of water at room temperature (22 ± 2 °C)—filled (1.9 L) and open (3.8 L) circles and triangles (50 ± 2 °C). (**a**) Tests with freshly formulated stones; and (**b**) test with a stone after several months use in water with a temperature of about 45–55 °C. Each cycle before 401 was done for 6 h; (**c**) Results of antimicrobial tests with stones.

The stones formulated in this study were capable of releasing small, but relatively uniform, quantities of copper ions into water over a period of several months (Figure 3a,b). The amount of copper dissolving into water produced concentrations of 0.05 to 0.20 mg/L in 24 to 72 h immersion times. The supply of copper into water became more uniform after the first few days (Figure 3a) and remained practically unchanged even after one year of the stone’s use for water disinfection, ~400 cycles of leaching tests (Figure 3b). These small quantities of copper ions are sufficient to disinfect bacteria contaminated water. It should be noted that the same ceramics released 10 to 20 times more copper to water before copper ion reduction to sub-microparticles (not shown).

In standard antimicrobial tests, as per ASTM E2149-1 standard, clay-based ceramic decorated with copper sub-microparticles demonstrated a 1.5 Log kill of *Staphylococcus aureus* and 1.3 Log kill of *Klebsiella pneumoniae* at 1 h (95–97% reduction) (Figure 3c). Almost a complete kill of both organisms (>99.9%) happened after 3 h of contact time. A 6.83 Log kill of *Staphylococcus aureus* and 4.18 Log kill of *Klebsiella pneumoniae* at 5 h (>99.99% reduction) was also observed.

## 3. Materials and Methods

Pottery clay (Red Earthenware, Minnesota Clay Company, Plymouth, Minnesota, United States of America composed of mainly kaolin (55–80 wt %), silica (25–55 wt %) and talc (5–15 wt %) was used to shape 21 approximately fist-sized stones with diameters of 45–60 mm (Figure 1a). The stones were allowed to sit and dry in atmospheric conditions overnight. The clay stones were then placed in a dryer at 100 °C for 2 days. Dried samples were fired in a furnace at 1000 °C for 2 h. Once cooled, the ceramic samples were then submerged in 1.73 M copper sulfate for 5 days at room temperature. After 5 days, the stones were removed from the solution and washed with tap water, and dried again. Copper ions introduced to ceramics were reduced to metallic copper sub-microparticles in a hydrogen atmosphere at 450 °C for 4 h. After cooling any excess residue was vacuumed and gently wiped from the surface of the samples before further testing.

Thin sections were cut from the ceramic stones using a diamond saw blade and then carbon coated. Scanning electron microscopy (SEM) imaging was done using a JEOL JSM-6400 (JEOL, Peabody, MA, USA) using 20 kV accelerating voltage. Elemental analysis of selected samples was done under SEM using energy dispersive spectroscopy (EDS).

X-ray diffraction (XRD) was performed on ceramic disc sections as well as a powdered form of the original clay using a Scintag XDS2000 powder diffractometer (Scintag Inc., Cupertino, CA, USA). A range of 2.00–70.00° was used with a step of 0.020° and a scan rate of 1.00°/min.

Selected ceramic stones were submerged in tanks containing 3.79 liters and 1.90 liters of tap water at either 20 or 50 °C (±2 °C). Water samples were taken from the tanks approximately every 24 h on the weekdays and 72 h over the weekend. After each sample, the tanks were emptied, and new tap water was added to re-submerge the stones. This continued for 35 days with 24 samples taken, with the stones being submerged in water for approximately 840 h total. In another durability test, stones were used for 4–6 days a week for approximately 48 weeks out of the year. The stones remained in warm water (45–55 °C) for ~6 h a day.

Antimicrobial tests were done on freshly formulated stones after they were pre-washed with water several times to secure uniform release of copper ions. *Staphylococcus aureus* ATCC 6538 and *Klebsiella pneumoniae* ATCC 4352 were grown in individual overnight cultures of TSB at 37 °C. The overnight cultures were used to spike test vessels for ASTM E2149-1 testing as indicated. ASTM E2149-1 was used for antimicrobial activity determination with the following modifications. Briefly, 2.835 liters of sterile, distilled deionized water was placed within a 5 liter Erlenmeyer flask, sealed with foil and incubated overnight at 49 °C to prewarm the testing solution to levels that are seen in massage clinics that use prewarmed stones for massage. One sterile stone was placed in each flask and 50 milliliters of overnight culture was added to each test flak in quintuplicate. There was one control flask and stone per organism type. The starting concentrations of viable organism per flask was determined to be 6.83 Log for *Staphylococcus aureus* and 5.60 Log for *Klebsiella pneumoiae* via heterotrophic plate count at the 0 h time point. Flasks were shaken as per ASTM E2149-1 and one milliliter samples were removed from the flasks at 0, 1, 3, 5 and 24 h post inoculation and plated on Tryptic Soy Agar, incubated for 24–48 h at 37 °C and enumerated. Calculation of log kill was performed using the following formula:

Log Reduction = log10 (N/T),
(4)

where N is the number of viable organisms in the control group (without copper) at time point x, and T is the number of viable organisms in the treatment group (exposed to active-containing product) at time point x.

## 4. Conclusions

A novel antimicrobial stone was formulated, characterized and tested in this study. Clay-based porous ceramic was functionalized with copper sub-microparticles through a copper ion adsorption and exchange processes and reduction of ionic copper to metallic copper using hydrogen. The stone had a gradient distribution of copper sub-microparticles, with the highest copper content in the stone surface region (~7 wt %) and <1 wt % in the internal structure at a depth of ~5 mm. Water leached copper from stones in quantities of 0.05–0.20 mg/L in tests with 1.9–3.8 L water and immersion times of 24 to 72 h. A year-long test demonstrated long-term durability of stones expressed through uniform and consistent release of copper to water. Copper content remained at 0.05–0.20 mg/L in ~4 L water after 6 h immersion over >400 cycles. These small quantities of released copper make the stone a powerful disinfectant of bacteria-contaminated water. This novel antimicrobial ceramic demonstrated a 1.5 Log kill of *Staphylococcus aureus* and 1.3 Log kill of *Klebsiella pneumoniae* at 1 h (95–97% reduction) and an almost complete kill of both organisms (>99.9%) at 3 h of contact time. A 6.83 Log kill of *Staphylococcus aureus* and 4.18 Log kill of *Klebsiella pneumoniae* was achieved at 5 h (>99.99% reduction).

These results suggest copper-infused ceramic to be a promising antibacterial product for water disinfection, and potentially other future commercial and domestic applications.
